# Clinicopathological characteristics of adrenal tumors in older adults: a cohort study

**DOI:** 10.3389/fendo.2026.1852735

**Published:** 2026-07-07

**Authors:** Yuanyuan Tang, Mou Peng, Rongrong Cui, Yunliang Gao

**Affiliations:** 1 Department of Oncology, The Second Xiangya Hospital, Central South University, Changsha, Hunan, China; 2 Department of Urology, The Second Xiangya Hospital, Central South University, Changsha, Hunan, China; 3 National Clinical Research Center for Metabolic Diseases, Changsha, Hunan, China; 4 Department of Metabolism and Endocrinology, The Second Xiangya Hospital of Central South University, Changsha, Hunan, China; 5 National Clinical Research Center for Mental Disorders, Changsha, Hunan, China; 6 Center for Adrenal Disorders, The Second Xiangya Hospital, Central South University, Changsha, Hunan, China

**Keywords:** adrenal tumor, malignant, metastasis, older adults, predictor

## Abstract

**Introduction:**

To investigate age-specific characteristics of adrenal tumors in older adults and to identify predictors for adrenal malignancy.

**Methods:**

Inpatients ≥60 years old with adrenal tumor evaluated at a large medical center between January 2011 and December 2023 were retrospectively enrolled.

**Results:**

Of 2,633 inpatients with adrenal tumor, 559 (21.2%, ≥60 years) were eligible for analysis, 52.8% were men, with a median age of 64 years. 58.7% patients were incidentally diagnosed. The median tumor size was 29 mm, and the median unenhanced computed tomographic (CT) attenuation was 15.0 Hounsfield units (HU). Older patients presented a relatively high prevalence of malignant adrenal tumors, accounting for 19.0% of all cases. Adrenal metastases were the most common malignant adrenal tumors, accounting for 59.4% of all malignant cases. A risk model was conducted to predict adrenal malignancy, and the area under the curve was 0.89. Predictors included incidental discovery, male, bilateral tumor, older age, higher HU, and lower weight. The optimal cutoff value was age=67.5 years, size=42.5 mm and CT attenuation=20.5 HU, respectively.

**Conclusions:**

Older patients presented the age-specific spectrum of diagnosis, in particular a high prevalence of adrenal metastases. Certain predictors may help to differentiate a malignant lesion from a benign one. This study helps to expand the current knowledge of the adrenal tumor in older adults and inspire robust therapies with better outcomes.

## 1 Introduction

Adrenal tumors are frequently encountered in clinical practice, with the majority being discovered incidentally on imaging studies ordered for indications unrelated to suspected adrenal pathology (1, 2). The advent and widespread availability of cross-sectional imaging have significantly increased the detection of these tumors compared with previous eras. Notably, the prevalence of adrenal tumors rises with advancing age, peaking between the fifth and seventh decades of life (3). As shown by a population-based study, the overall prevalence of adrenal tumors was 532 per 100,000 (0.53%) inhabitants, with the highest incidence observed in patients older than 65 years (2). Radiological series have documented a prevalence ranging from 2.1% to 5.1% in adults, with rates increasing to approximately 5%–10% in individuals over 70 years of age (4–7). Numerous autopsy series have consistently reported an overall prevalence ranging from 0.03% to 8.7%, and this prevalence is significantly elevated in individuals over the age of 50 (8–16). Therefore, the older population may suffer from a higher risk of adrenal tumors. Given the secular trend of population aging, older adults might be expected to constitute the largest age-group among patients with adrenal tumors in most countries. A high volume of these lesions could place a significant strain on healthcare systems.

Notably, adrenal tumors in older patients often exhibit a clinical presentation that differs from younger counterparts, influencing diagnostic and therapeutic considerations. Younger patients with adrenal tumors show a stronger predisposition toward functional lesions and those associated with hereditary genetic syndromes (17). Instead, non-functional adrenal adenomas and malignant tumors appear to be more commonly observed in older adults (18, 19). Additionally, aging could contribute to the progressive impairment of physiological signaling mechanisms that determine lower incremental amplitude of secretion (20, 21). The signs and symptoms of adrenal hormone excess in older adults are frequently absent, subtle, or atypical and thus difficult to detect. Moreover, perioperative morbidity and mortality might be supposed to a higher risk of concomitant illnesses such as cardiovascular disease (CVD) (22, 23). Obviously, adrenal tumors in older patients pose specifically diagnostic and therapeutic challenges.

However, to our knowledge, there is only a paucity of studies focusing on adrenal tumor profiles in older adults (18, 19). The current study presented one of the largest pooled analyses of older patients with adrenal tumors, with an emphasis on the features of adrenal malignancies. The primary goal was to elucidate the age-specific traits of adrenal tumors in older adults, providing new evidence to foster the development of optimized diagnostic and therapeutic strategies for this group.

## 2 Subjects and methods

Inpatients ≥60 years old with adrenal tumor evaluated at our tertiary referral center between January 2011 and December 2023 were retrospectively enrolled. Those patients with a finding of adrenal tumor/mass in imaging studies (CT/MRI) were included. Charts were reviewed for demographic data. Tumor size was defined as the greatest diameter recorded on radiological imaging, with the priority of CT, MRI, and echo. The CT attenuation values was determined by an oval region-of-interest cursor.

Hormonal evaluation and diagnostic criteria: The functional status of adrenal tumors was retrospectively obtained through a comprehensive review of medical records. The biochemical workup mainly included the following: Cortisol excess was assessed using serum cortisol (8:00, 16:00, 24:00), 24-h urinary free cortisol, plasma adrenocorticotropic hormone (ACTH), and 1-mg dexamethasone suppression tests (DSTs). Supine and upright plasma aldosterone concentrations and renin activity were measured, and the plasma aldosterone-to-renin ratio (ARR) was calculated. Confirmatory tests for hyperaldosteronism involved a saline infusion and/or a captopril challenge test when available. Biochemical testing for pheochromocytoma was carried out using plasma-free metanephrine and normetanephrines, and urine vanillylmandelic acid (VMA). Other hormonal measurements for adrenal androgens were performed if deemed clinically necessary.

Surgical indications and clinical decision-making: Due to the retrospective nature of this 12-year study, the precise multidisciplinary decision-making processes could not be systematically retrieved for all patients. However, the indications for adrenalectomy were retrospectively ascertained from medical records and were primarily based on the following clinical and radiological criteria: (1) imaging features suspicious for malignancy, (2) biochemical evidence of hormone excess causing clinical symptoms, (3) significant tumor enlargement during follow-up, or (4) patient preference. The final decision for surgery was made after a comprehensive evaluation by the attending physicians or a multidisciplinary team (MDT) when available.

As previously described (2), adrenal tumors were grouped into four main diagnostic categories: (a) adrenal adenoma and nodular hyperplasia, (b) other benign tumors, (c) malignant tumors, and (d) pheochromocytoma. For patients who underwent adrenalectomy or adrenal biopsy, the diagnosis was definitively based on histopathology. For those who did not undergo surgical resection or biopsy, the final diagnosis was retrieved from the medical charts, which was primarily based on the integration of clinical manifestations and radiological characteristics at presentation and during follow-up.

Data were presented as median with interquartile range (IQR) for continuous values and as frequencies (percentages) for discrete ones. Student’s t-test, chi-square test, and Mann–Whitney test were used for statistical analysis as appropriate. A multivariable logistic regression was performed in GraphPad Prism 9 with malignancy (yes/no) as the binary outcome. All variables were defined *a priori* and entered simultaneously as main effects (forced entry); no stepwise selection was applied. Model output includes aOR (95% CI), AICc, Hosmer–Lemeshow goodness of fit, and the area under the receiver operating characteristic curve (ROC) AUC. Subjects with any missing value among selected predictors were excluded from the regression fit. An internal validation was performed by comparing AICc of the selected model vs. intercept-only model (AICc (intercept-only) − AICc (selected)=310.8−232.6 = 78.2], supporting predictive contribution over null).

Furthermore, an HU-based malignancy prediction model was developed in the subgroup of patients with radiologically indeterminate lesions, which we operationally defined as unenhanced CT attenuation >10HU. It was consistent with current ESE and AAES guidance where homogeneous lesions with HU ≤ 10 are regarded as benign-appearing (lipid-rich adenoma-like) and require no further dedicated adrenal imaging. The indeterminate (HU>10) stratum represents a particularly challenging decision zone in adrenal management.

All statistical analyses were performed by using GraphPad Prism9.0 (GraphPad Software, San Diego, CA, USA). A p<0.05 was considered as statistical difference. This study received approval from the local Ethics Committee of the Second Xiangya Hospital of Central South University (No. LYF2023076).

## 3 Results

### 3.1 Demographic characteristics

**Figure 1:**
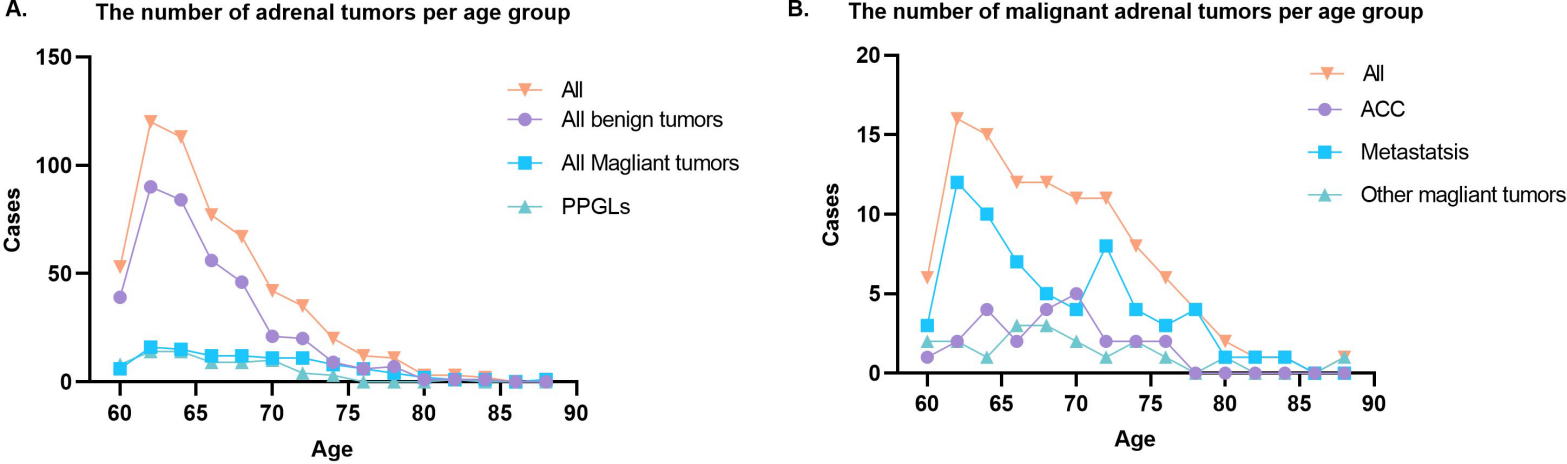
Number of adrenal tumor cases per age group in older patients. **(A)** The number of adrenal tumors in different groups (all benign ones, all malignant ones, and pheochromocytoma (PPGLs)). **(B)** Number of adrenal tumors in different groups (adrenocortical carcinoma (ACC), metastasis, and other malignancies.

A total of 2,633 inpatients with adrenal tumor were identified, and 559 (21.2%) patients (≥60 years old) were eligible for analysis (**Table 1**). These 559 patients were diagnosed at a median age of 64 (62–68) years, and 295 (52.8%) were men. The prevalence of adrenal tumor peaked at age 62 and declined thereafter (**Figure 1**). The median body weight was 60 (55–66) kg. A total of 150 (26.8%) patients were diagnosed with diabetes mellitus, 331 (59.2%) with hypertension, and 125 (22.4%) with CVD. Additionally, 59 (10.6%) patients had a history of alcohol consumption, and 140 (25.0%) patients had a smoking history.

**Table 1:** Demographic characteristics, clinical presentation, and management of older patients with adrenal tumors.

Variable	Total (n=559)
Age at diagnosis (years), median [IQR](range)	64 [62-68] (60-88)
Sex	
Men	295 (52.8%)
Women	264 (47.2%)
Body weight (kg), median [IQR] (range)	60 [54-66] (32-101)
Accompanying disease status	
Hypertension (isolated)	331 (59.2%)
DM (isolated)	150 (26.8%)
Cardiovascular disease	125 (22.4%)
History, no. (%)	
Smoking	140 (25.0%)
Alcohol consumption	59 (10.6%)
Mode of discovery, no. (%)	
Incidental	328 (58.7%)
Non-incidentally discovered (hormone excess, etc.)	176 (31.5%)
Voiding dysfunctions	11 (2.0%)
Others	44 (7.9%)
With lab hormonal test	445 (79.6%)
Non-functional tumors	203 (45.6%)
Aldosterone-secreting tumors	134 (30.1%)
Pheochromocytoma	65 (14.6%)
Cortisol-secreting tumors	37 (8.3%)
Others	6 (1.3%)
Location of adrenal tumor, no. (%)	
Left	290 (51.9%)
Right	216 (38.6%)
Bilateral	53 (9.5%)
Maximal mass size (mm), median [IQR] (range)	29 [18-55] (4-251.0)
Unenhanced CT attenuation (HU), median [IQR] (range)	15[2-32] (-96-260)

CT, computed tomography; DM, diabetes mellitus; HU, Hounsfield units. Data are presented as median [interquartile range] (range: minimum–maximum) for continuous variables.

Regarding the mode of discovery, most adrenal tumors (58.7%) were incidentally found. The distribution of tumors was as follows: 290 (51.9%) left-sided, 216 (38.6%) right-sided, and 53 (9.5%) bilateral. The median tumor size was 29 (18–55) mm, with a median attenuation value of 15 (2–32) HU on unenhanced CT scan.

### 3.2 Functional status of adrenal tumors

In all, 445 patients performed a lab hormonal test. Among them, 203 (45.6%) patients were classified as functionally active, whereas 242 (54.4%) were non-functional (**Table 1**). The distribution of specific hormone-secreting subtypes included 37 cases of cortisol-secreting tumors, 134 cases of aldosterone-secreting tumors, 65 cases of pheochromocytoma, and 6 cases of others.

Additionally, of the 328 patients with incidental lesions, 229 underwent laboratory hormonal evaluation, of whom 142 (62.0%) were clinically classified as nonfunctioning (**Table 2**).

**Table 2:** Demographic characteristics, clinical presentation, and management of patients with incidental or hormone-secreting adrenal tumors.

Variable	Incidental group (n=328)	Non-incidentally discovered group (n=176)	P value
Age at diagnosis (years), median [IQR] (range)	62 [62-69] (60-84)	64 [62-67] (60-83)	0.001
Sex			>0.05
Women	150 (45.7%)	85 (48.3%)	
Men	178 (54.3%)	91 (51.7%)	
Body weight (kg), median [IQR] (range)	59 [54-66] (32-101)	62.5 [57-70] (37-95)	0.002
Accompanying disease status			
Hypertension (isolated)	152 (46.3%)	151 (85.8%)	<0.001
DM (isolated)	80 (24.4%)	57 (32.4%)	>0.05
Cardiovascular disease	59 (18.0%)	51 (29.0%)	0.004
History, no. (%)			
Smoking	82 (25.0%)	46 (26.1%)	>0.05
Drinking	32 (9.8%)	24 (13.6%)	>0.05
With lab hormonal test	229 (69.8%)	173 (98.3%)	<0.001
Non-functional tumors	142 (62.0%)	42 (24.3%)	
Pheochromocytoma	32 (14.0%)	25 (14.5%)	
Aldosterone-secreting tumors	34 (14.8%)	90 (52.0%)	
Cortisol-secreting tumors	17 (7.4%)	16 (9.2%)	
Others	4 (1.7%)	0	
Location of adrenal tumor, no. (%)			>0.05
Left	168 (51.2%)	100 (56.8%)	
Right	132 (40.2%)	66 (37.5%)	
Bilateral	28 (8.5%)	10 (5.7%)	
Maximal mass size (mm), median [IQR] (range)	33 [21-63] (4-251)	21 [15-36] (7-130)	<0.001
Unenhanced CT attenuation (HU), median [IQR] (range)	20[2-34] (−94-260)	10 [2-22] (−96-110)	>0.05
Adrenalectomy, no. (%)	167 (50.9%)	140 (79.5%)	<0.001

CT, computed tomography; DM, diabetes mellitus; HU, Hounsfield units. Data are presented as median [interquartile range] (range: minimum–maximum) for continuous variables.

### 3.3 Malignancy assessment and final diagnoses

Of the 559 tumors, 106 (19.0%) were ultimately diagnosed as malignant, 381 (68.2%) as benign, and 72 (12.9%) as pheochromocytomas. Among the 106 malignant tumors, 63 (59.4%) were metastatic lesions, 24 (22.6%) were ACC, 10 (9.4%) were lymphomas, and 9 (8.5%) were other rare malignancies.

**Figure 2:**
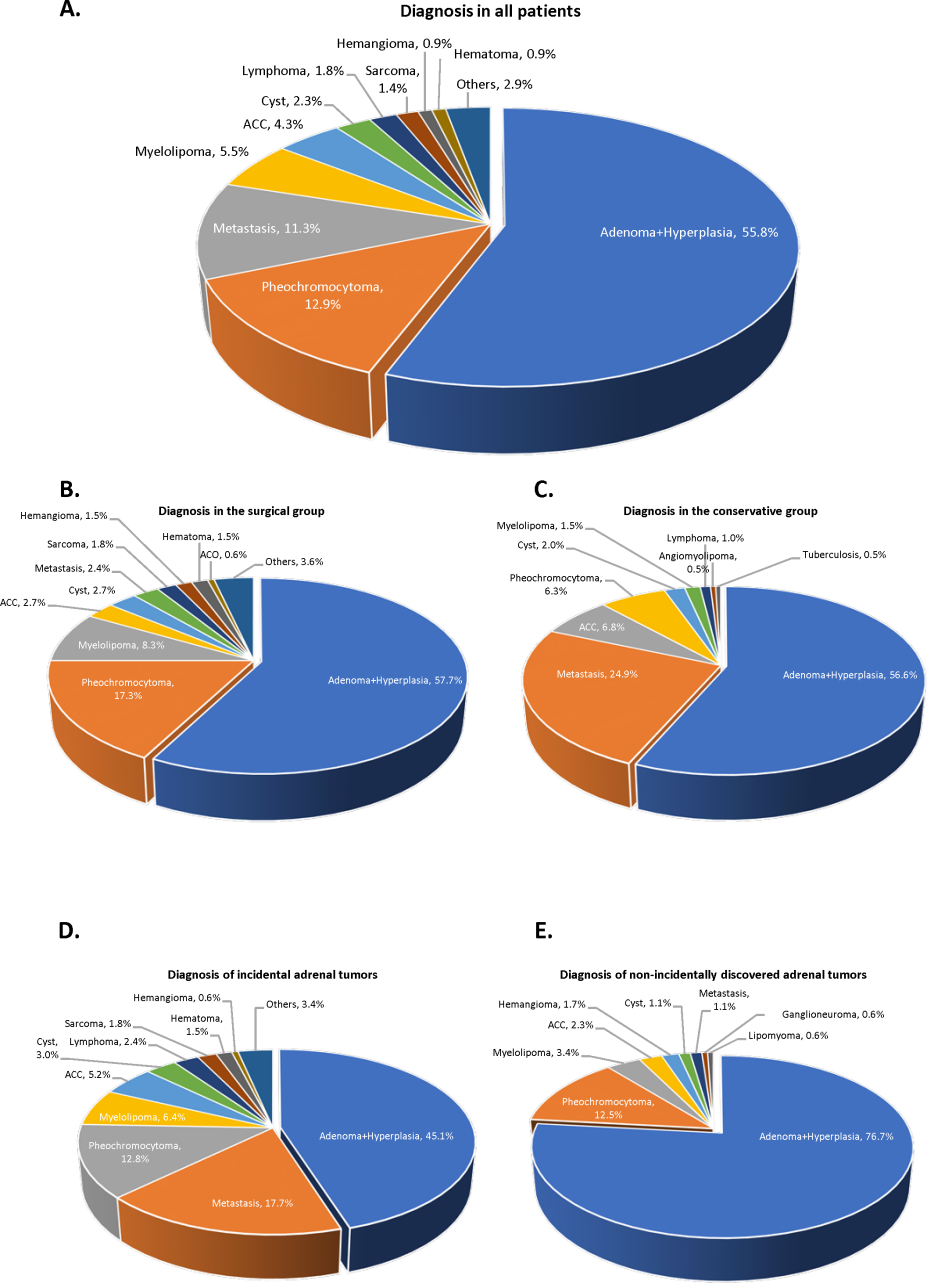
The diagnosis of adrenal tumors in older patients. **(A)** Diagnosis in all patients. **(B)** Diagnosis in patients with surgery treatment. **(C)** Diagnosis in patients with conservative treatment. **(D)** Diagnosis in patients with incidental adrenal tumors. **(E)** Diagnosis in patients with non-incidentally discovered adrenal tumors. Tumor types ranking outside the top 10 in proportion were grouped into the “Other” category. ACC, adrenocortical carcinoma; ACO, adrenocortical oncocytoma.

Among 559 patients, the most commonly encountered diagnosis of benign lesions was adrenal adenoma and nodular hyperplasia (55.8%), consisting of adenoma (45.4%) and hyperplasia (10.4%). Additionally, 12.3% of patients were diagnosed with other types of benign adrenal tumor such as myelolipoma (5.5%), cyst (2.3%), and so on (**Figure 2**, **Table 3**). The prevalence of almost all adrenal tumor subtypes reached its peak at 62 years of age and then steadily declined (**Figure 1**).

**Table 3:** The diagnosis of adrenal tumors in older patients.

Types	Total (n=559)		Surgical group (n=336)		Conservative group (n=223)			Incidental group (n=328)		Non-incidentally discovered group (n=176)		
	N	%	N	%	N	%	P	N	%	N	%	P
Adenoma and nodular hyperplasia	312	55.8	194	57.7	118	52.9	<0.001	148	45.1	135	76.7	<0.001
Adenoma	254	45.4	157	46.7	97	43.5		131	39.9	101	57.4	
Hyperplasia	58	10.4	37	11	21	9.4		17	5.2	34	19.3	
Other benign tumors	69	12.3	60	17.9	9	4	<0.001	48	14.6	13	7.4	<0.001
Myelolipoma	31	5.5	28	8.3	3	1.3		21	6.4	6	3.4	
Cyst	13	2.3	9	2.7	4	1.8		10	3.0	2	1.1	
Hemangioma	5	0.9	5	1.5	0	0		2	0.6	3	1.7	
Hematoma	5	0.9	5	1.5	0	0		5	1.5			
ACO	2	0.4	2	0.6	0	0		1	0.3			
Angiomyolipoma	2	0.4	1	0.3	1	0.4		1	0.3			
Ganglioneuroma	2	0.4	2	0.6	0	0		1	0.3	1	0.6	
Inflammation	2	0.4	2	0.6	0	0		2	0.6			
Lipomyoma	2	0.4	2	0.6	0	0		1	0.3	1	0.6	
Lymphangioma	2	0.4	2	0.6	0	0		1	0.3			
Leiomyoma	1	0.2	1	0.3	0	0		2	0.6			
RHE	1	0.2	1	0.3	0	0		1	0.3			
Tuberculosis	1	0.2	0	0	1	0.4						
Malignant tumors	106	19	24	7.1	82	36.8	<0.001	90	27.4	6	3.4	<0.001
Metastasis	63	11.3	8	2.4	55	24.7		58	17.7	2	1.1	
ACC	24	4.3	9	2.7	15	6.7		17	5.2	4	2.3	
Lymphoma	10	1.8	1	0.3	9	4		8	2.4			
Sarcoma	8	1.4	6	1.8	2	0.9		6	1.8			
Adenocarcinoma	1	0.2	0	0	1	0.4		1	0.3			
Pheochromocytoma	72	12.9	58	17.3	14	6.3	<0.0001	42	12.8	22	12.5	>0.05

ACC, adrenocortical carcinoma; ACO, adrenocortical oncocytoma; RHE, retiform hemangioendothelioma.

### 3.4 Comparison between incidentally and non-incidentally discovered adrenal tumor

Patients with incidental tumors were significantly younger (62 (62–69) vs. 64 (62–67)), with less comorbidity related to hypertension (46.3% vs. 85.8%) and CVD (18.0% vs. 29.0%), and lower body weights (59 (54–66) vs. 62.5 (57–70) kg) (**Table 2**). Incidental adrenal tumors were generally larger (33 (21–63) vs. 21 (15–36) mm) and exhibited higher unenhanced CT attenuation (20 (2–34) vs. 10 (2–22) HU), whereas surgical resection was less common (50.9% vs. 79.5%). No significant difference was found in sex and tumor location between groups.

Furthermore, a subgroup analysis between incidentally and non-incidentally discovered adrenal tumor demonstrated that non-incidentally discovered ones were most commonly diagnosed as adrenal adenoma and nodular hyperplasia (76.7% vs. 45.1%), but less identified as malignant (3.4% vs. 27.4%) or other benign tumors (7.4% vs. 14.6%) (**Table 3**).

### 3.5 Comparison between surgical and conservative treatments

Surgery was performed in 336 (60.1%) patients. Patients being younger (64 (62–67) vs. 66 (63–71) years old), female (53.0% vs. 38.6%), with hypertension (66.4% vs. 48.4%), but without CVD (18.5% vs. 28.3%) or smoking history (19.4% vs. 33.4%) would be preferentially recommended to surgery (**Table 4**). The surgical group had a significantly higher prevalence of hormone-secreting adrenal tumor (41.7% vs. 16.1%). Tumors that were larger (median: 30 mm), were unilateral, and exhibited low HU values (median: 12) were more likely to undergo surgical resection. A higher rate of incidental tumors was noted in the conservative group (72.2% vs. 49.7%). Two groups showed no significant differences in other demographic characteristics.

**Table 4:** Demographic characteristics of older patients with surgery or conservative management.

Variable	Surgical group (n=336)	Conservative group (n=223)	P value
Age at diagnosis (years), median [IQR] (range)	64 [62-67] (60-82)	66 [63-71] (60-88)	<0.01
Sex			<0.01
Women	178 (53.0%)	86 (38.6%)	
Men	158 (47.0%)	137 (61.4%)	
Body weight (kg), median [IQR] (range)	60 [55-66] (32-101)	60 [53-67] (37-90)	>0.05
Accompanying disease status			
Hypertension (isolated)	223 (66.4%)	108(48.4%)	<0.01
DM (isolated)	85 (25.3%)	65 (29.2%)	>0.05
Cardiovascular disease	62 (18.5%)	63(28.3%)	<0.01
History, no. (%)			
Smoking	65 (19.4%)	75(33.4%)	<0.01
Alcohol consumption	31(9.2%)	28 (12.6%)	>0.05
Mode of discovery, no. (%)			<0.001
Incidental	167 (49.7%)	161 (72.2%)	
Non-incidentally discovered (hormone excess, etc.)	140 (41.7%)	36 (16.1%)	
Voiding dysfunctions	9 (2.7%)	2 (0.9%)	
Others	20 (6.0%)	24 (10.8%)	
With lab hormonal test	285 (84.8%)	160 (71.8%)	<0.05
Location of adrenal tumor, no. (%)			<0.01
Left	185 (55.0%)	105 (47.1%)	
Right	138 (41.1%)	78 (35.0%)	
Bilateral	13 (3.9%)	40 (17.9%)	
Maximal mass size (mm), median [IQR] (range)	30[20-59] (9-251)	23[15-50] (4-210)	<0.01
Unenhanced CT attenuation (HU), median [IQR] (range)	12[0-30] (−96-170)	19[5-34] (−80-260)	<0.05

CT, computed tomography; DM, diabetes mellitus; HU, Hounsfield units. Data are presented as median [interquartile range] (range: minimum–maximum) for continuous variables.

A subgroup analysis between patients with or without surgery revealed that the surgical group presented a significant higher proportion of adrenal adenoma and nodular hyperplasia (57.7% vs. 52.9%), pheochromocytoma (17.3% vs. 6.3%), and other benign tumors (17.9% vs. 4.0%), but a lower proportion of malignant tumors (7.1% vs. 36.8%) (**Table 3**).

### 3.6 Demographic characteristics of patients with different adrenal tumors

Patients with different types of adrenal tumors exhibited significant disparities in demographic features (**Table 5**). Those with malignant adrenal tumors were significantly oldest, were predominantly men (76.4%), and had the highest smoking rates (39.6%) but lowest prevalence of diabetes (17.9%) and hypertension (34.9%). Incidental detection was common across benign (51.4%), malignant (84.9%), and pheochromocytoma (58.3%) cases, yet malignancies were the most frequent incidental finding. Bilateral adrenal tumors were most frequently diagnosed to be malignant. Pheochromocytomas had the largest tumor sizes (58 (19-184) mm) and highest CT attenuation values (39.5 (−10-50) HU).

**Table 5:** Demographic and clinical characteristics of older patients with different types of adrenal tumors.

Variable	Total (n=559)	Benign tumor (n=381)	Malignancy (n=106)	Pheochromocytoma (n=72)	P value
Age at diagnosis (years), median [IQR] (range)	64[62-68] (60-88)	64[62-67] (60-83)	67[60-72] (60-88)	64.5[62-69] (60-82)	<0.001
Sex					<0.001
Men	295 (52.8%)	175 (45.9%)	81 (76.4%)	39 (54.2%)	
Women	264 (47.2%)	206 (54.1%)	25 (23.6%)	33 (45.8%)	
Body weight (kg), median [IQR] (range)	60 [54-66] (32-101)	61 [55-69] (32-101)	59 [53-63] (40-80)	56 [51-65] (37-84)	<0.001
Accompanying disease status					
Hypertension (isolated)	331 (59.2%)	253 (66.4%)	37 (34.9%)	41 (56.9%)	<0.001
DM (isolated)	150 (26.8%)	104 (27.3%)	19 (17.9%)	27 (37.5%)	<0.05
Cardiovascular disease	125 (22.4%)	92 (24.1%)	17 (16.0%)	16 (22.2%)	>0.05
History, no. (%)					
Smoking	140 (25.0%)	81 (21.3%)	42 (39.6%)	17 (23.6%)	<0.001
Alcohol consumption	59 (10.6%)	40 (10.5%)	12 (11.3%)	7 (9.7%)	>0.05
Mode of discovery, no. (%)					<0.001
Incidental	328 (58.7%)	196 (51.4%)	90 (84.9%)	42 (58.3%)	
Non-incidentally discovered (hormone excess, etc.)	176 (31.5%)	148 (38.9%)	6 (5.7%)	22 (30.6%)	
Voiding dysfunctions	11 (2.0%)	10 (2.6%)	1 (0.9%)	0 (%)	
Others	44 (7.9%)	27 (7.1%)	9 (8.5%)	8 (11.1%)	
With lab hormonal test	445 (79.6%)	336 (88.2%)	42 (39.6%)	67 (93.1%)	<0.001
Location of adrenal tumor, no. (%)					<0.001
Left	290 (51.9%)	206 (54.1%)	51 (48.1%)	33 (45.8%)	
Right	216 (38.6%)	146 (38.3%)	31 (29.2%)	39 (54.2%)	
Bilateral	53 (9.5%)	29 (7.6%)	24 (22.6%)	0 (0%)	
Maximal mass size (mm), median [IQR] (range)	29 [18-55] (4-251)	24[16-38] (4-251)	43 [24-73] (9-143)	58[40-78] (19-184)	<0.001
Unenhanced CT attenuation (HU), median [IQR] (range)	15 [2-32] (−96-260)	9 [−2-20] (−96-260)	30.5[22-37] (−24-171)	39.5 [32-44] (10-50)	<0.001

CT, computed tomography; DM, diabetes mellitus; HU, Hounsfield units. Data are presented as median [interquartile range] (range: minimum–maximum) for continuous variables.

A further subgroup analysis identified significant differences in demographic characteristics of patients with different types of malignant adrenal tumor (**Supplementary Table 1** in **Supplementary 1**). Patients with metastasis had the lowest rate of diabetes mellitus (7.9%), followed by ACC (12.5%) and other malignancies (57.9%). ACC, metastasis, and other malignancies were predominantly incidentally detected (70.8%, 92.1%, 78.9%, respectively), with metastasis exhibiting the highest detection rate among these groups. In all, the median size of malignant adrenal tumors was over 40 mm and ACC presented the largest size (75.0 (15-140) mm), followed by other malignancies (73.0 (24-143) mm) and metastasis (28.0 (9-100) mm).

### 3.7 Predictors of malignant adrenal tumors

On multivariable logistic regression, incidental mode of discovery (odds ratio [OR], 6.47 [95% CI, 2.47 to 19.50]), male sex (OR, 6.35 [95% CI, 2.27 to 19.09]), bilaterally located tumor (OR, 6.15 [95% CI, 2.25 to 17.78]), older age (OR, 1.08 [95% CI, 1.00 to 1.16]), and higher unenhanced CT attenuation (OR, 1.02 [95% CI, 1.01 to 1.03]) were independently associated with increased odds of malignancy (**Table 6**). Conversely, higher body weight was associated with a lower odds of malignancy (OR, 0.94 [95% CI, 0.89 to 0.98]). The AUC of risk model was 0.89 [95% CI, 0.85 to 0.93].

**Table 6:** Univariate and multivariate logistics analyses—predictors of a malignant adrenal tumor (malignancies n=106).

	Univariate analysis	Multivariate analysis		
Variable	P value	Odds ratio	95% CI	P value
Mode of discovery (incidental vs. non-incidental)	<0.001	6.47	2.47 to 19.50	<0.001
Sex (male vs. female)	<0.001	6.35	2.27 to 19.09	<0.001
Location (bilateral vs. unilateral)	<0.001	6.15	2.25 to 17.78	<0.001
Age at diagnosis	<0.001	1.08	1.00 to 1.16	=0.05
Unenhanced CT attenuation (HU)	<0.001	1.02	1.01 to 1.03	<0.001
Body weight	=0.001	0.94	0.89 to 0.98	<0.01
Hypertension (yes vs. no)	<0.001			>0.05
DM (yes vs. no)	<0.05			>0.05
Smoking (yes vs. no)	<0.001			>0.05
Maximal mass size (mm)	<0.001			>0.05

A dedicated prediction model for malignancy was developed in the subgroup of patients with indeterminate lesions (unenhanced HU >10) (**Table 7**). In multivariable analysis, bilaterally located tumor (OR, 3.45 [95% CI, 1.23 to 10.03]), male sex (OR, 2.76 [95% CI, 1.09 to 7.21]), and older age (OR, 1.13 [95% CI, 1.04 to 1.23]) were related to higher malignancy odds. Instead, hypertension was related to a lower odds of malignancy in this model (OR, 0.45 [95% CI, 0.20 to 0.97]). The model achieved an AUC of 0.83 [95% CI, 0.77 to 0.90].

**Table 7:** Univariate and multivariate logistics analyses—predictors of a malignant adrenal tumor in patients with HU>10 (malignancies n=53).

	Univariate analysis	Multivariate analysis		
Variable	P value	Odds ratio	95% CI	P value
Location (bilateral vs. unilateral)	<0.01	3.45	1.23 to 10.03	<0.05
Sex (male vs. female)	<0.001	2.76	1.09 to 7.21	<0.05
Age at diagnosis	<0.001	1.13	1.04 to 1.23	<0.01
Hypertension (yes vs. no)	<0.001	0.45	0.20 to 0.97	<0.05
Mode of discovery (incidental vs. nonincidental)	<0.001			>0.05
Maximal mass size (mm)	<0.01			>0.05
Smoking (yes vs. no)	<0.01			>0.05

Additionally, a subgroup of patients with available lab hormonal test demonstrated that bilaterally located tumor (OR, 19.70 [95% CI, 3.88 to 120.60]), incidental mode of discovery (OR, 4.76 [95% CI, 1.15 to 25.24]), and tumor size (OR, 1.04 [95% CI, 1.02 to 1.06]) were positively correlated with malignant adrenal tumors (**Supplementary Table 2** in **Supplementary 1**). In contrast, CVD (OR, 0.04 [95% CI, 0.00 to 0.37]) showed a correlation with reduced odds of malignancy. The AUC of the risk model was up to 0.91 [95% CI, 0.85 to 0.97].

**Figure 3:**
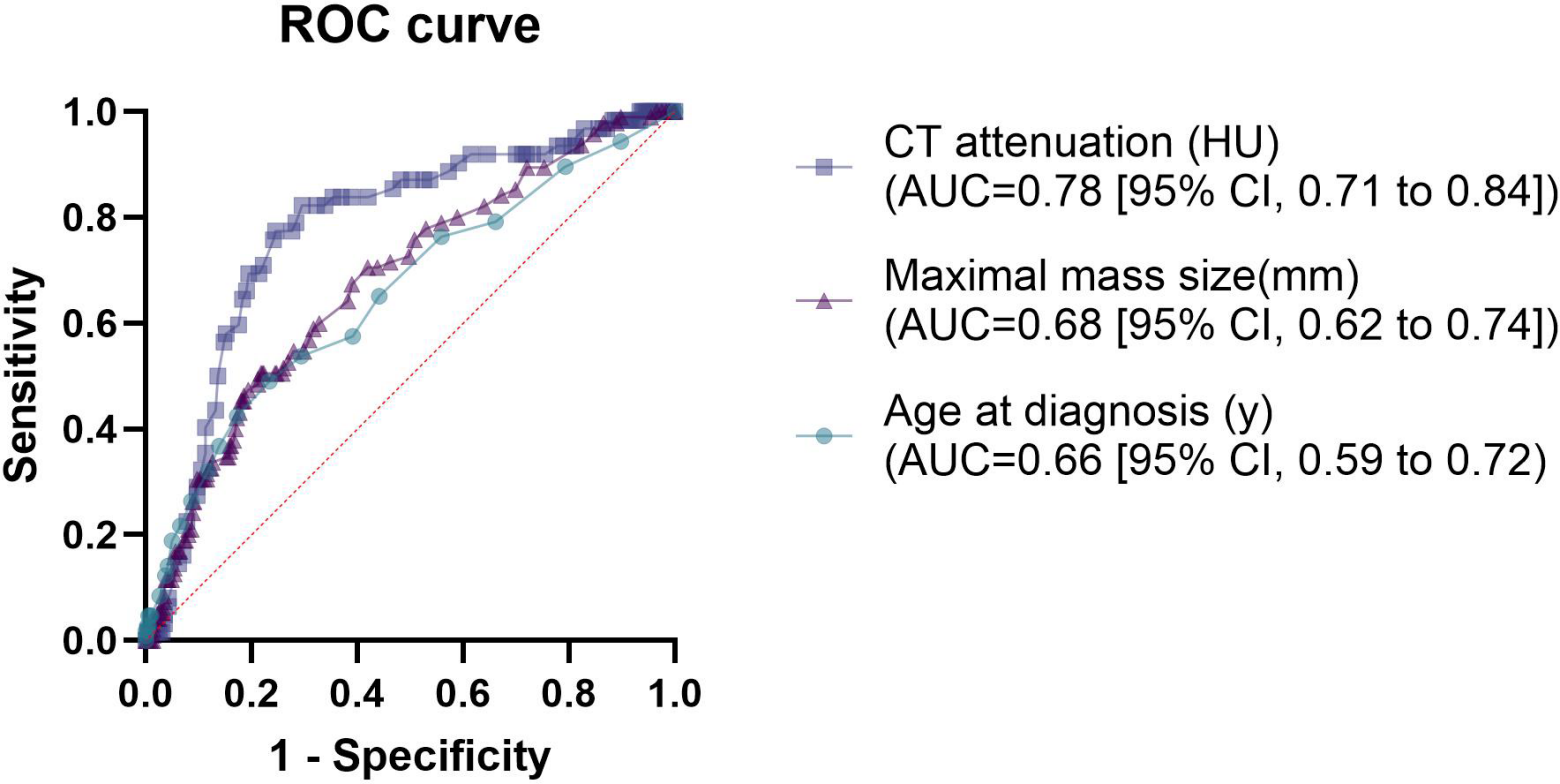
ROC curve of the selected risk factors responsible for the malignant adrenal tumors in older patients. HU, Hounsfield units.

Furthermore, we conducted an ROC analysis for age at diagnosis, tumor size, and unenhanced CT attenuation, with AUC values of 0.66 [95% CI, 0.59 to 0.72], 0.68 [95% CI, 0.62 to 0.74], and 0.78 [95% CI, 0.71 to 0.84], respectively. The optimal cutoff values identified were 67.5 years old (sensitivity: 49.1%; specificity: 76.6%) for age at diagnosis, 42.5 mm (sensitivity: 50.5%; specificity: 78.2%) for tumor size, and 20.5 HU (sensitivity: 77.4%; specificity: 75.5%) for unenhanced CT attenuation, respectively (**Figure 3**).

## 4 Discussion

Our study was one of the largest pooled analyses of adrenal tumors in older patients and provided further insights into the characteristics of these tumors. Older patients displayed an age-specific spectrum of diagnoses, with a high prevalence of malignant adrenal tumors, notably metastases. Given the necessity to assess both hormonal function and malignant potential in newly diagnosed adrenal tumors, this study provided a detailed analysis of predictive factors for malignancy and construct concomitant risk models. Additionally, diagnostic and therapeutic challenges still existed possibly due to unique clinical presentation, age-related physiological changes, and the overlapping of tumor-related symptoms by comorbidities. Through the present study, we hope to expand the current knowledge of the adrenal tumor in older adults and inspire robust therapies with better outcomes.

### 4.1 Unique clinical presentation

#### 4.1.1 Mode of discovery

In our study, the majority of adrenal tumors (58.7%) were discovered incidentally rather than hormone excess suspicion. The proportion of adrenal incidentaloma here was slightly lower than that in the general population, where previous studies showed 67% to 82.0% of adrenal tumor were incidentally diagnosed (2, 24, 25). This shortfall of incidentaloma could be explained by tertiary referral filtering. As a tertiary referral center for complex adrenal and endocrine/oncology cases, our institution is intrinsically enriched for symptomatic, larger, or oncologically relevant lesions. Correspondingly, those small, clearly benign-appearing lipid-rich adenomas detected incidentally in the community are more likely managed conservatively and not referred to a tertiary setting. Therefore, our 58.7% incidental share may reflect a referral-filtered case mix, not a community prevalence.

Additionally, our study revealed a relatively high proportion of functional tumors among adrenal lesions initially classified as incidentalomas. This is an interesting and potentially important finding, possibly representing a true clinical characteristic or reflecting differences in classification or diagnostic approach in older population. This phenomenon may stem from methodological variability and biological plausibility. First, advances in assay sensitivity and evolving clinical guidelines over a 12-year period may have facilitated the detection of subtle hormonal hypersecretion that would have previously gone unnoticed. Therefore, part of this observed prevalence may reflect improved diagnostic capability. Second, symptomatic “clues” of adrenal hormone secretion in older adults are frequently attributed to aging, polypharmacy, or comorbidity. As a result, hormone-secreting lesions may first surface as imaging-noticed rather than symptom-driven, which can raise the apparent “functional ones” among incidentals. Future studies are necessary to explore this observation.

#### 4.1.2 Sex asymmetry

Our study presented a male predominance in adrenal tumors in older adults. This finding contradicted prior research about a female predominance in general population (26–31). Higher proportions of adrenal malignancies and pheochromocytoma in our male patients may lead to the contradiction (**Table 5**). In addition, men may be more susceptible to age-related structural changes in male adrenal glands such as a reduction in cortical zonation width, possibly hampering the diagnostic progress (32). Therefore, a more careful evaluation should be made when facing a male patient with adrenal tumor.

#### 4.1.3 Age-related comorbidities

A high prevalence of comorbidities was found in our older patients, with 59.2% having hypertension, 26.8% diabetes mellitus, and 22.4% CVD. Several studies have also reported that older patients with adrenal tumors are at a higher risk for these comorbidities compared with younger patients or general population (33–36). These comorbidities can complicate the clinical presentation and mask symptoms of functional adrenal tumors, delaying appropriate diagnosis and treatment. As reported in our study, approximately 39.9% older patients received conservative treatment, particularly those being older, male, with CVD and smoking history.

### 4.2 Age-specific spectrum of adrenal tumors

#### 4.2.1 Malignant adrenal tumors in older adults

In our cohort, malignant adrenal tumors accounted for 19.0% of cases, aligning closely with rates (10.5%-19.6%) reported in previous single-center studies of older adults (18, 37). However, studies involving the general population indicated that the prevalence of malignant adrenal tumor varied from 4.5% to 8.7% (2, 24, 30). Our results indicated that adrenal metastases were the predominant malignant entity, comprising a majority (59.4%) of the malignant adrenal tumors in this study. This translated to an overall prevalence of adrenal metastases of 11.3% (63/559), a rate considerably higher than that reported in prior studies. For instance, prior studies have reported lower prevalence rates of adrenal metastases in older adults, such as 1.1% in a population-based study of patients over 60 (2) and 5.8% in a study focused on adrenal incidentalomas in older adults (19). Therefore, older patients appear to face a greater risk of developing malignant adrenal tumors in particular metastases, with an occurrence rate exceeding previous estimates. Moreover, a key finding of our study was that the majority of malignant adrenal tumors, including metastases, were discovered incidentally. One possible explanation is that older patients are more likely to have incidental detection of adrenal metastases during imaging for unrelated issues or during cancer staging. Instead, younger patients often present with symptomatic metastases leading to their diagnosis (38). Currently, there was lack of specific guidelines for the older adults, and the diagnosis could only be referred to several published guidelines about management of adrenal incidentaloma and other types of adrenal tumors (39, 40). Based on the significant disparities in clinical characteristics identified in this study, we propose that malignancy should be strongly considered in older, male patients or those presenting with bilateral adrenal tumors larger than 40 mm in diameter. In addition, those older patients with adrenal incidentalomas who are not surgical candidates require vigilant follow-up, as the risk of metastatic disease increases significantly in those with a known oncologic history (41).

Following metastases, ACC was the second most prevalent malignant subtype, accounting for 4.3% of total adrenal tumors. This was quite similar to previous studies only involving older patients, which reported a rate ranging from 4% to 7% (18, 37). ACC here was frequently discovered incidentally and notably, presented with a larger tumor size compared with adrenal metastases. One possible explanation is that adrenal metastasis may be discovered at an earlier stage due to regular follow-up in those patients with a previous malignant tumor history.

However, accurately differentiating malignant adrenal tumors from benign ones remains a significant clinical challenge. Several studies have estimated the risk factors of malignant adrenal tumors, but their findings are primarily derived from the general population (25, 42). We firstly explored the demographic predictors for malignant adrenal tumors in older patients and identified male sex, older age at diagnosis, bilaterally located tumor, incidental mode of discovery, lower body weight, and higher unenhanced CT attenuation as independent risk factors. The AUC of our risk model reached up to 0.89. Additionally, we structured the analysis in patients with radiologically indeterminate or non-adenomatous lesions represents the most challenging and clinically relevant scenario in real-world practice. The AUC of this HU-based risk model reached up to 0.83. Our model may provide an individualized malignancy probability that can be acted upon in precisely this indeterminate-imaging scenario—informing the MDT decision between close imaging surveillance, further characterization, and adrenalectomy. Moreover, for those with hormonal test, bilaterally located tumor, CVD, tumor size, and incidental mode of discovery were predictors and the AUC of risk model could go up to 0.91. Consequently, our risk models may facilitate clinicians to estimate the likelihood of malignancy in an older patient.

Given the pivotal role of age, tumor size, and radiologic features in clinical decision-making, we further refined and explored the diagnostic value of these three predictors to distinguish benign and malignant adrenal tumors. We found that an unenhanced CT attenuation cutoff of more than 20 HU had the highest sensitivity. Commonly, the 10 HU threshold on unenhanced CT is accepted to distinguishing benign from malignant adrenal masses. However, a previous study indicated that 20 HU had similar sensitivity but higher specificity for malignancy (25), which was consistent with our study. Additionally, the cutoff value for tumor size was 42.5 mm in our study, closely aligned with other studies that reported that a tumor size threshold over 40 mm effectively distinguished ACC from benign adrenal tumors with a high sensitivity but a relatively low specificity (26, 43). Moreover, we firstly defined a cutoff value of 67.5 years old for age to distinguish between benign and malignant ones. However, this age cutoff had relatively low sensitivity (49.1%) and specificity (76.6%) and should better be applied in conjunction with tumor size, imaging characteristics, and other predictors. Collectively, multiple cutoff values are recommended to combine to capture the variability in tumor characteristics and improve diagnostic accuracy in older patients.

#### 4.2.2 Adrenocortical adenoma in older adults

Our study indicated that the most type of adrenal tumor in older patients was adrenal adenoma and nodular hyperplasia (55.8%). It was consistent with previous studies reporting a rate of 43%-56.7% in older patients (18, 37). In addition, we found that 50.0% of adrenal adenoma with hormonal testing were non-functional (**Supplementary Table 3** in **Supplementary 1**). Therefore, the decision to proceed with adrenal surgery in older adults should be more careful. Furthermore, a large part of adrenal adenomas in this study was adrenal incidentaloma. The characteristics of adrenal incidentalomas have been explored by numerous studies, providing valuable guidance for their diagnosis and treatment (1, 39, 44).

### 4.3 Limitations

Despite the strengths of this study, certain limitations should be addressed including a single-center retrospective design, some parts of missing data, the use of body weight instead of BMI, and the limited number of the total population. Firstly, the primary limitation of this study is its retrospective nature and the extended 12-year study period. Consequently, the diagnostic workup, hormonal evaluation protocols, and the specific clinical guidelines referenced evolved considerably over time. This resulted in inherent methodological heterogeneity across the cohort, meaning that a completely unified diagnostic criterion could not be strictly applied to all patients. In addition, a plenty of patients with adrenal tumor did not receive a complete hormonal workup or were clinically diagnosed without histology. Those with hormonal evaluation frequently could not give detailed test data. The final diagnosis of adrenal tumor was retrieved from the medical charts for those without surgical resection or biopsy. Furthermore, due to the variability in historical medical documentation, the precise multidisciplinary decision-making processes leading to adrenalectomy could not be systematically ascertained for every case. Under this condition, our conclusions may not accurately reflect the true situations. Therefore, our recommendations should be further verified and carefully applied in clinical practice. Despite these constraints, we believe our findings provide valuable real-world insights into the longitudinal management of adrenal tumors in older adults. Secondly, our study used 60 instead of 65 years old as the cutoff for defining older adults, mainly due to alignment with regional standards and the demographic context of the population in China. Thirdly, a limitation of our prediction model is the use of body weight instead of BMI. We acknowledge that body weight alone does not adequately reflect adiposity and is heavily influenced by confounders common in older adults, such as frailty, sarcopenia, and disease-related weight loss (cachexia). Therefore, the observed association between lower body weight and higher malignancy risk may be driven by underlying paraneoplastic processes or nutritional decline rather than representing an independent physiological risk factor. Clinicians should interpret this variable cautiously within the context of the patient’s overall clinical picture. Fourthly, although our study represented one of the largest cohorts of older patients with adrenal tumors to date, the sample size was still relatively limited and lack of comparison with younger patients. Moreover, we only included older patients without younger counterparts, possibly reducing the confidence power. Fifthly, a tertiary referral selection bias may exist in our study. Our institution is a large medical center functioning as a tertiary referral center for complex adrenal and endocrine/oncology cases. As results, our cohort is enriched for larger, more suspicious, or oncological lesions, and the observed malignancy prevalence cannot be generalized to the population-wide prevalence of adrenal tumors. This referral-filtering effect is well described in the literature and should be kept in mind when interpreting our absolute malignancy rate. However, we believe that our findings are still comparable with similar research in particular early published studies.

## 5 Conclusions

Our present study explored the clinicopathological context of adrenal tumors in older adults and highlighted a high prevalence of malignant adrenal tumors in particular metastases. A risk model was developed to differentiate a malignant lesion from a benign one. This study may expand the current knowledge of adrenal tumor in older adults, increase the awareness of malignant tumor diagnosis, and inspire careful evaluation for a better outcome. Further investigations are necessary to refine adrenal tumor diagnostic strategies, explore novel predictors of malignancy, and determine personalized treatment models for this population.

## Data Availability

The original contributions presented in the study are included in the article/**Supplementary Material**. Further inquiries can be directed to the corresponding authors.
