# Supplementary material for: Clinicopathological characteristics of adrenal tumors in older adults: a cohort study
Source: Front Endocrinol (Lausanne). 2026 Jul 7;17:1852735. doi: 10.3389/fendo.2026.1852735 (PMC13385050; doi:10.3389/fendo.2026.1852735)
Supplement: Supplementary file 1 [file Table1.docx]

**Supplements:**

**Table S1.** Demographic and clinical characteristics of patients with malignant adrenal tumors

**Table S2.** Univariate and multivariate logistics analyses–predictors of a malignant adrenal tumor with available work-up for adrenal glucocorticoid production (n=42)

**Table S3.** Adrenocortical adenoma with hormonal test

**Table S1. Demographic and clinical characteristics of patients with malignant adrenal tumors**

| Variable | All (n=106) | ACC(n=24) | Metastasis (n=63) | Other malignancies (n=19) | P value |
| --- | --- | --- | --- | --- | --- |
| Age at diagnosis (y), median [IQR] (range) | 67 [64-72](60-88) | 68 [64-71] (60-76) | 66 [63-72] (60-84) | 67 [63-73] (60-88) | >0.05 |
| Sex |  |  |  |  | >0.05 |
| Women | 25 (23.6%) | 8 (33.3%) | 11 (82.5%) | 6 (31.6%) |  |
| Men | 81 (76.4%) | 16 (66.7%) | 52 (17.5%) | 13 (68.4%) |  |
| Body weight (kg), median (range) | 59 [53-63](40-80) | 58[53-62] (41-80) | 60[53-64] (40-80) | 55.5 [52-62](46-70) | >0.05 |
| Accompanying disease status |  |  |  |  |  |
| DM (isolated) | 19 (17.9%) | 3 (12.5%) | 5 (7.9%) | 11 (57.9%) | 0.006 |
| Hypertension (isolated) | 37 (34.9%) | 10 (41.7%) | 18 (28.6%) | 9 (47.4%) | >0.05 |
| Cardiovascular disease | 17 (16.0%) | 3 (12.5%) | 12 (19.0%) | 2 (10.5%) | >0.05 |
| History, No. (%) |  |  |  |  |  |
| Drinking | 12 (11.3%) | 3 (12.5%) | 5 (7.9%) | 4 (21.1%) | >0.05 |
| Smoking | 42 (39.6%) | 9 (37.5%) | 26 (41.3%) | 7 (36.8%) | >0.05 |
| Mode of discovery, No. (%) |  |  |  |  | 0.014 |
| Incidental | 90 (84.9%) | 17 (70.8%) | 58 (92.1%) | 15 (78.9%) |  |
| Non-incidentally discovered (hormone excess, etc.) | 6 (5.7%) | 4 (16.7%) | 2 (3.2%) | 0 (0%) |  |
| Voiding dysfunctions | 1 (0.9%) | 1 (4.2%) | 0 (0%) | 0 (0%) |  |
| Others | 9 (8.5%) | 2 (8.3%) | 3 (4.8%) | 4 (21.1%) |  |
| With lab hormonal test | 42 (39.6%) | 19 (79.2%) | 11 (17.5%) | 12 (63.2%) | <0.001 |
| Location of adrenal tumor, No. (%) |  |  |  |  | >0.05 |
| Left | 51 (48.1%) | 13 (54.2%) | 34 (54.0%) | 4 (21.1%) |  |
| Right | 31 (29.2%) | 8 (33.3%) | 17 (27.0%) | 6 (31.6%) |  |
| Bilateral | 24 (22.6%) | 3 (12.5%) | 12 (19.0%) | 9 (47.4%) |  |
| Maximal mass size(cm), median (range) | 43 [24-73] (9-143) | 75 [50-86] (15-140) | 28 [18-36] (9-100) | 73 [58-100] (24-143) | <0.001 |
| Unenhanced CT attenuation (HU), median (range) | 30.5 [22-37] (-24-171) | 35 [24-39] (15-43) | 30 [18-34] (-12-171) | 33 [27-40] (-24-41) | >0.05 |

Abbreviations: CT, computed tomography; DM, diabetes mellitus; HU, Hounsfield units. Footnote:​ Data are presented as Median [Interquartile Range] (Range: Minimum–Maximum) for continuous variables.

**Table S2. Univariate and multivariate logistics analyses–predictors of a malignant adrenal tumor with available work-up for adrenal glucocorticoid production (n=42)**

|  | Univariate analysis |  | Multivariate analysis | | |
| --- | --- | --- | --- | --- | --- |
| Variable | P value |  | Odds ratio | 95% CI | P value |
| Sex (male vs female) | <0.01 |  |  |  | >0.05 |
| Age at diagnosis | <0.05 |  |  |  | >0.05 |
| Location (bilateral vs unilateral) | <0.01 |  | 19.70 | 3.88 to 120.60 | <0.01 |
| Mode of discovery (incidental vs nonincidental) | <0.001 |  | 4.76 | 1.15 to 25.24 | <0.01 |
| Maximal mass size (mm) | <0.001 |  | 1.04 | 1.02 to 1.06 | <0.001 |
| Cardiovascular disease (yes vs no) | <0.01 |  | 0.04 | 0.00 to 0.37 | <0.01 |
| Body weight | <0.05 |  |  |  | >0.05 |
| Hypertension (yes vs no) | <0.001 |  |  |  | >0.05 |
| Unenhanced CT attenuation (HU) | <0.05 |  |  |  | >0.05 |

Abbreviations: CT, computed tomography; HU, Hounsfield units.

**Table S3. Adrenocortical adenoma with hormonal test**

| Types | Total (n=226) | |  | Surgical group (n=134) | |  | Conservative group (n=92) | |  |  | Incidental group (n=86) | |  | Hormone-secreting group (n=98) | |  |
| --- | --- | --- | --- | --- | --- | --- | --- | --- | --- | --- | --- | --- | --- | --- | --- | --- |
|  | N | % |  | N | % |  | N | % | P |  | N | % |  | N | % | P |
| Nonfunctional Tumors | 113 | 50 |  | 38 | 28.4 |  | 75 | 81.5 | <0.0001 |  | 67 | 77.9 |  | 21 | 21.4 | <0.001 |
| Cortisol-Secreting Tumors | 19 | 8.4 |  | 13 | 9.7 |  | 6 | 6.5 |  |  | 6 | 7 |  | 12 | 12.2 |  |
| Aldosterone-Secreting Tumors | 90 | 39.8 |  | 80 | 59.7 |  | 10 | 10.9 |  |  | 12 | 14 |  | 63 | 64.3 |  |
| Others | 4 | 1.8 |  | 3 | 2.2 |  | 1 | 1.1 |  |  | 1 | 1.2 |  | 2 | 2 |  |
